# Identifying target areas of medicines information efforts to pregnant and breastfeeding women by reviewing questions to SafeMotherMedicine: A Norwegian web-based public medicines information service

**DOI:** 10.1186/s12884-022-05252-3

**Published:** 2022-12-02

**Authors:** Tina Bakkebø, Kristine Heitmann, Kamilla Vågsvoll, Hilde Erdal, Jan Schjøtt

**Affiliations:** 1 Regional Medicines Information and Pharmacovigilance Centre (RELIS Vest), Department of Clinical Biochemistry and Pharmacology, Haukeland University Hospital, Bergen, Norway; 2 Department of Clinical Science, Faculty of Medicine, University of Bergen, Bergen, Norway; 3 Department of Global Public Health and Primary Care, University of Bergen, Bergen, Norway

**Keywords:** Non-prescription drugs, Prescription drugs, Medicines information, Teratology information services, Pregnancy, Breastfeeding, Risk perception, Information need, Internet

## Abstract

**Background:**

Online information about safety of medications during pregnancy and breastfeeding is shown to be conflicting, resulting in anxiety and abstaining from use. The aim of this study was to characterize questions to SafeMotherMedicine, a web-based medicines information service for pregnant and breastfeeding women, to identify target areas that could guide subsequent development of medicines information directed at pregnant and breastfeeding women.

**Methods:**

The SafeMotherMedicine database contains all questions received through the web-based service and their corresponding answers. A retrospective database analysis of questions received from January 2016 to September 2018 was performed, using descriptive statistics.

**Results:**

A total of 11 618 questions were received including 5 985 questions (51.5%) concerning pregnancy, 4 878 questions (42.0%) concerning breastfeeding, and 755 questions (6.5%) concerning both conditions. The medications in question represented all therapeutic groups with paracetamol (7.0%), ibuprofen (4.1%), cetirizine (3.3%), desloratadine (3.2%) and meclizine (2.8%) being the top five. The 20 medications most frequently asked about for either pregnancy, breastfeeding or both pregnancy and breastfeeding, constituted half of all questions and were used to identify target areas. These included both symptomatic relief of common complaints, such as pain, nausea, and rhinitis, as well as treatment of chronic conditions such as allergy, psychiatric disorders, and asthma. Analysis of a subset of questions showed that most of these questions were asked before use of medications in a current pregnancy (49%) or during breastfeeding (72%). The questions concerned use of medications in all stages of pregnancy and breastfeeding. For 81.6% of the questions concerning pregnancy, and for 84.2% of the questions concerning breastfeeding, information of no or low risk for the foetus or the breastfed infant was provided by SafeMotherMedicine.

**Conclusions:**

We found that target areas for medicines information directed at pregnant and breastfeeding women included both symptomatic relief of common complaints as well as treatment of chronic conditions. The questions concerned a wide range of medications and involved use in all stages of pregnancy and breastfeeding. Our findings indicate that developing medicines information addressing the identified target areas will meet the information need for a large proportion of this patient group.

**Supplementary Information:**

The online version contains supplementary material available at 10.1186/s12884-022-05252-3.

## Background

Use of medications among pregnant and breastfeeding women is common. An international study found that approximately 80% of pregnant women use medications [1], while the prevalence among breastfeeding women is reported to be from 30–100% [2, 3]. For many conditions that can occur during pregnancy, such as urinary tract infection, asthma, or severe depression, it is well established that the benefit of using medications outweighs the potential risk for the foetus [4–6]. However, pregnant women commonly overestimate the risk of using medications [7, 8], which may lead to untreated illness that could adversely affect maternal and foetal health [9]. Also during breastfeeding, women are reported to be concerned about use of medications in fear of harming the breastfed child [2, 3], despite the fact that most medications do not readily cross over to breastmilk in significant amounts [10]. Thus, temporary or permanent cessation of breastfeeding due to maternal use of medications, is rarely considered necessary.

The internet has been shown to be an important source for pregnant women seeking information about safety on use of medications [11, 12]. However, online information has been found to be conflicting and inconsistent [13, 14], resulting in anxiety and abstaining from use of the medication [15]. We know that pregnant women’s beliefs and risk perception influence their therapeutic decision making [16]. To promote appropriate use of medications, pregnant and breastfeeding women need access to medicines information that provide balanced information on safety, benefit, and risk. Patient-specific information provided by medicines information centres and teratogen information services have been found to influence therapeutic decisions among pregnant and breastfeeding women [17–19]. Identifying therapeutic fields where there is a large information need among pregnant and breastfeeding women could be used to guide future development of medicines information. Analysing frequently asked questions from pregnant and breastfeeding women to medicines information services provides a knowledge basis for identifying areas for such targeted medicines information.

SafeMotherMedicine is a web-based medicines information service directed towards pregnant and breastfeeding women. The service is run by the Regional Medicines Information and Pharmacovigilance Centre (RELIS), a Norwegian network of medicines information centres [20]. RELIS is staffed by pharmacists and physicians with expertise in searching and critical evaluation of literature, as well as risk communication. As 13% of nearly 3000 enquires yearly from health care professionals to RELIS concern use of medications during pregnancy or breastfeeding [21], the staff at RELIS has acquired extensive experience with searching for and providing information on teratology and safety in breastfeeding. Among physicians in Norway, RELIS has been ranked highest as a source of information on use of medications during pregnancy and breastfeeding [19]. SafeMotherMedicine was established in 2011 based on the idea that a national web-based question and answer service directed towards pregnant and breastfeeding women would relieve the health care service.

The aim of this study is to identify target areas that could guide subsequent development of medicines information directed at pregnant and breastfeeding women, based on a review of questions to SafeMotherMedicine.

## Methods

### Study material

In SafeMotherMedicine, all questions received through the web-based service and their corresponding answers are stored in a full-text database, searchable by staff only. The inquirer index the question with the relevant condition (e.g. pregnancy, breastfeeding, or both pregnancy and breastfeeding). The inquirer is also asked to describe medications, indication, dosage and duration of treatment, previous communication with physician, level of breastfeeding (exclusive or partial), and if the breastfeed child is healthy, sick, or premature. The medications in question are indexed by staff according to the Anatomical Therapeutic Chemical (ATC) classification system for medications [22] before the question and answer pair is stored in the database. In the ATC system, medications are classified in groups at five different levels, where the ATC 1^st^ level has 14 main anatomical or pharmacological groups, and the ATC 5^th^ level denotes the chemical or generic substance [22].

### Study protocol

A retrospective, descriptive analysis of the SafeMotherMedicine database from January 2016 to September 2018 was performed. Only questions concerning conventional medications from the pharmaceutical industry were included, while questions concerning food, dietary supplements, cosmetics, and complementary therapies were excluded. The 20 medications most frequently asked about in the categories; pregnancy, breastfeeding and both pregnancy and breastfeeding, were categorized into therapeutic fields, that pointed out the target areas. This categorization was based on presumed indication, which again was based on ATC code as well as the authors’ experience from SafeMotherMedicine.

A subset of 550 questions was randomly selected from the total material using Research Randomizer [23]. The size of the subset was based on time constraints with regard to analysis, as well as what was considered to be sufficient for the purpose. The subset was analysed by the authors through a combination of text inspection and evaluation of the associated indexation in the database. The questions and their corresponding answers were examined for any further description of:

- Why the question was raised (Second opinion, use discouraged by health care professionals or product information or conflicting information, general question (including those with no reason given for raising the question), other (e.g. correct dosage, duration of treatment), symptoms in nursing child).
- When the question was submitted relative to use of the medication(s) in question (During planning of pregnancy or breastfeeding, before use in women already pregnant or breastfeeding, during use, after use, other (e.g. paternal use or occupational exposure), not described).
- Intended use of the medication(s) in question relative to stage of pregnancy or age of breastfed child (Possibly pregnant, 1^st^ trimester, 2^nd^ trimester, 3^rd^ trimester/ 0-2 weeks, 2 weeks – 6 months, 6–12 months, > 12 months, not pregnant/breastfeeding yet, not described).
- Which information of teratogenic risk/risk for breastfed child was provided (No or low risk, possibly increased or increased risk, unknown risk/sparse documentation, information on risk not provided).
- Advice that was given (Can be used, should not be used, non-classifiable answer).

Each question could involve several medications, and each medication was assessed individually.

### Statistics

Data for questions to SafeMotherMedicine was collected by Structured Query Language (SQL), transferred to Excel, and subsequently analysed with statistical software SPSS version 25 (IBM Corp, Armonk, NY). For the subset of questions that was studied in more detail, a pilot study was performed based on the 30 initial questions in the study material. Seven questions were excluded from the pilot material because they did not concern medications. The remaining 23 questions concerned 29 medications. In the pilot study, consistency among the authors with regard to classification of questions was considered as excellent with an Intraclass Correlation Coefficient (ICC) of 0.97 for questions concerning pregnancy (n = 15), and 0.92 for questions concerning breastfeeding (n = 17), both p < 0,0001.

## Results

In the study period, SafeMotherMedicine received 11 618 questions including 5 985 (51.5%) concerning pregnancy, 4 878 (42.0%) concerning breastfeeding, while 755 questions (6.5%) concerned both conditions. Excluding 1 084 (9.3%) questions that did not concern medications, a mean of 1.48 medications per question was observed. Sixty-four percent (63.7%) of the questions concerned only 1 medication, and the number of indexed medications in a question ranged between 1–9. Mean number of medications was comparable in questions concerning pregnancy (1.50), breastfeeding (1.43), and both conditions (1.65).

The medications were distributed among all ATC-groups, with medications used for disorders related to the respiratory and nervous system being the most frequent. Top five medications were paracetamol (7.0%), ibuprofen (4.1%), cetirizine (3.3%), desloratadine (3.2%) and meclizine (2.8%). With regard to the main ATC-groups, questions concerning either pregnancy, breastfeeding or both pregnancy and breastfeeding had a comparable pattern of distribution, as illustrated in Supplementary Figure 1.

**Fig. 1:**
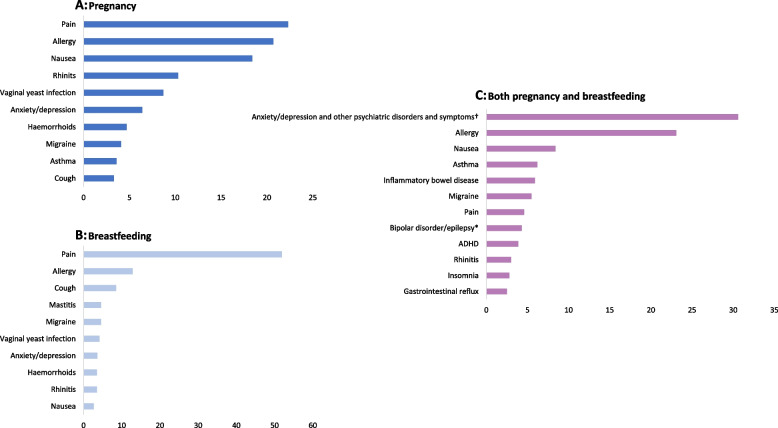
Therapeutic fields most frequently asked about during A: Pregnancy (*n* = 2 979), B: Breastfeeding (*n* = 2 316), and C: Both pregnancy and breastfeeding (*n* = 442). The top 20 medications for questions concerning use during the respective categories A-C are sorted into their corresponding therapeutic fields (y-axis) according to their relative frequency (%) among the top 20 medications (x-axis). Categorization of medications into therapeutic fields was based on presumed indication, which again was based on ATC code as well as the authors’ experience from SafeMotherMedicine. For complete overview of medications involved in Fig. 1 and their categorization in therapeutic fields, see Supplementary Tables 1, 2, 3. Data based on questions to SafeMotherMedicine from January 2016 to September 2018. † This category includes antidepressants, benzodiazepines and quetiapine *This category includes solely lamotrigine

Figure 1 shows the therapeutic fields of the 20 medications most frequently asked about in the categories; pregnancy, breastfeeding and both pregnancy and breastfeeding, respectively. For all three categories together, this sums up to 36 medications that constitute half (49.3%) of all questions to SafeMotherMedicine in the study period. Top three medications concerning pregnancy were paracetamol (7.4%), meclizine (4.0%) and cetirizine (4.0%). Top three medications concerning breastfeeding were ibuprofen (7.3%), paracetamol (7.2%) and diclofenac (3.4%). Top three medications concerning use during both pregnancy and breastfeeding were escitalopram (6.6%), desloratadine (5.6%) and quetiapine (4.6%). For complete overview of medications involved in Fig. 1 and their categorization in therapeutic fields, see Supplementary Tables 1, 2, 3.

**Fig. 2:**
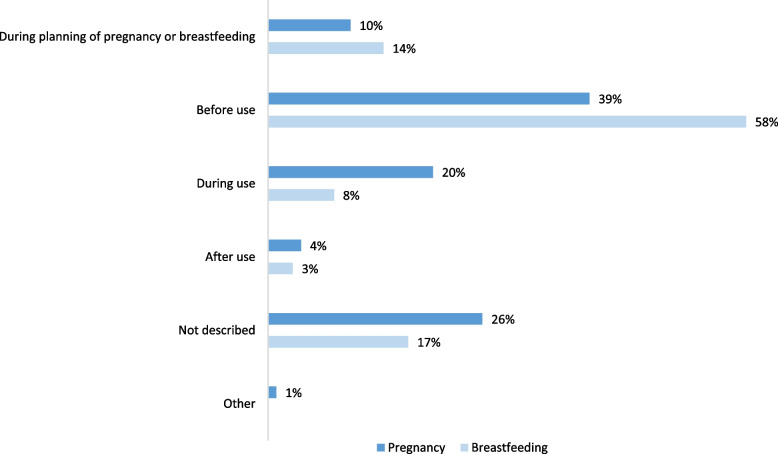
Submission of questions relative to use of medications during pregnancy and breastfeeding. Distribution of questions according to whether they were submitted before, during or after use of the medications in pregnancy (*n* = 300), or breastfeeding (*n* = 266). The “Other” category included three questions that did not concern maternal use of medications, but for instance paternal use and occupational exposure. Data based on a review of questions to SafeMotherMedicine from January 2016 to September 2018

Of a subset of 550 questions analysed in more detail, 138 questions were excluded, as they did not address use of specific medications, resulting in a remaining sample of 412 questions concerning in total 521 medications. Three hundred medications (57.6%) concerned pregnancy and 266 breastfeeding (51.1%). The majority of the questions associated with pregnancy involved women that asked about medications before use, or during use, in a current pregnancy (Fig. 2). Similarly, the questions associated with breastfeeding were most often asked before use in current breastfeeding period, or during planning of breastfeeding.

**Fig. 3:**
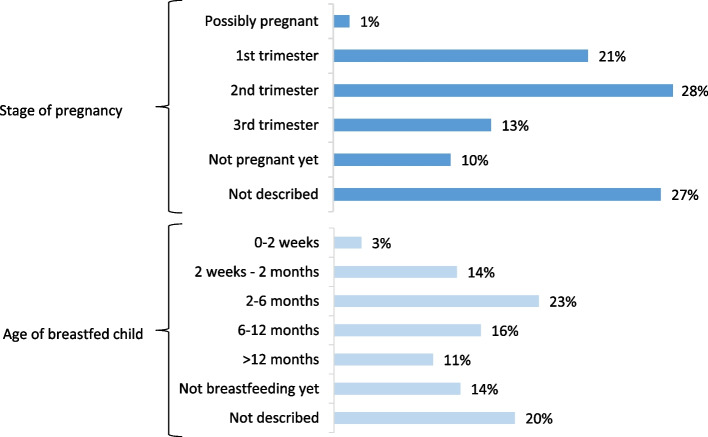
Stage of pregnancy or age of breastfed child relative to intended medication use. Distribution of questions according to at which stage of pregnancy (upper half of figure, *n* = 300) or age of breastfed child (lower half of figure, *n* = 266) the women intend to use the medication(s) in question. Data based on a review of questions to SafeMotherMedicine from January 2016 to September 2018

Questions to SafeMotherMedicine concerned use of medications in all trimesters of the pregnancy and use in all stages of breastfeeding (Fig. 3). For pregnancy, most questions concerned use in the first two trimesters, and for breastfeeding, most questions concerned use while breastfeeding infants up to 6 months of age.

For 237 (79.0%) of questions regarding pregnancy, and for 211 (79.3%) questions regarding breastfeeding, no specific reason was given for the why the questions were raised to SafeMotherMedicine. In 23 (8%) of the questions concerning pregnancy, and in 28 (11%) of the questions concerning breastfeeding, the reason for raising the question to SafeMotherMedicine was that use of the medication was discouraged by health care professionals or product information, or that the information in other sources was conflicting. For all these questions, SafeMotherMedicine gave reassuring advice and did not advise avoidance. No pattern was found with regard to which medications that were associated with conflicting information on safety.

For 245 of the 300 questions (81.6%) concerning pregnancy, SafeMotherMedicine provided information of no or low teratogenic risk, and for 224 of the 266 (84.2%) questions concerning breastfeeding, SafeMotherMedicine provided information of no or low risk for the breastfed infant. SafeMotherMedicine advised avoidance in 10 of 300 (3.0%) questions regarding pregnancy and in 8 of 266 (2.6%) regarding breastfeeding.

## Discussion

To the best of our knowledge, this study is the first analysis of questions and their corresponding answers on use of medications from both pregnant and breastfeeding women received through a web-based service. We found that complaints such as pain, nausea, and rhinitis, as well as chronic conditions such as allergy, psychiatric disorders, and asthma are involved in a significant amount of the questions, making them potential target areas to guide subsequent development of medicines information. The questions in the study concerned all stages of pregnancy and breastfeeding and reassuring advice was provided in most cases.

### Frequently asked about medications, therapeutic fields and target areas

Half of all questions were constituted by the 20 medications most frequently asked about for each of the categories; pregnancy, breastfeeding and both pregnancy and breastfeeding, that together with the medications’ corresponding therapeutic field pointed out the target areas. The identified target areas include complaints well known to be prevalent among pregnant and breastfeeding women, in addition to those known to pose a risk if undertreated. These findings imply that by directing information efforts towards these target areas, tailored to apply to all stages of pregnancy or breastfeeding, the information need can be met for many pregnant and breastfeeding women. However, questions concerning medications from a wide range of therapeutic fields were received, indicating an information need that cannot be covered by information directed at target areas only.

Medications used to treat complaints from the nervous system or respiratory system constitute a major part of the questions in our data material, including several medications also available over-the-counter (OTC), in line with previous studies [24–28]. Despite OTC medications generally being recognized as safe and available without involvement from a physician, our results support previous findings indicating a need among women to ensure safety of use during pregnancy and breastfeeding [27, 28]. OTC-medications in Norway are mainly available from pharmacies, but a few are also available from grocery shops, convenience stores and petrol stations. As use of these medications are prevalent among women of childbearing age [1], efforts to improve provision of evidence-based information about OTC medications in commonly used sources is needed.

### Information need according to condition

There were differences in the therapeutic fields most frequently asked about between the three categories pregnancy, breastfeeding and both pregnancy and breastfeeding (Fig. 1). This is not unexpected, as different complaints related to pregnancy and breastfeeding typically occur at different stages; e.g., nausea and vomiting, reflux and haemorrhoids are typical pregnancy complaints, whereas mastitis is associated with breastfeeding. However, it is noteworthy that nausea is the third most frequently asked about therapeutic field both in the category for women who ask about use in pregnancy and in the category for women who ask about use in both pregnancy and breastfeeding. This may indicate that some Norwegian women are breastfeeding into their next pregnancy. Guidelines for treatment of nausea and vomiting during pregnancy exist [29], however, little is found in the literature addressing treatment of this complaint in women that are still breastfeeding. Thus, questions about use of antiemetics may be raised due to uncertainty among the women themselves and health care professionals.

For use of medications during both pregnancy and breastfeeding, the high prevalence of questions concerning psychiatric disorders could be explained by planning of therapy due to chronic disease. This have also been shown by Pijpers et al., who found that use of psychotropic medications, in particular antidepressants, were important drivers to seek information [25]. Psychiatric diseases may require treatment throughout the pregnancy and breastfeeding period, and untreated psychiatric illness in the perinatal period carries risks to the mother, the foetus and infant [6, 30]. In addition, psychiatric medications are shown to cause extra concern and uncertainty among pregnant women compared to medications used for somatic disorders [18]. A study from Australia also indicated that women with psychiatric illness are more proactive and seek advice when planning a pregnancy, compared to other women [31]. Altogether, our results indicate that medications used in psychiatric diseases are a target area for information efforts directed at pregnant and breastfeeding women.

### When do the women ask for advice?

We found that women in most cases asked prior to use of a medication during pregnancy (67%) and breastfeeding (87%), reflecting a need of decision support prior to treatment initiation. This is similar to the findings from a North American Teratology Information Service (TIS), which reported that 80% of questions were asked before use of a medication during pregnancy or breastfeeding [24]. Breastfeeding is an expected condition, which enables planning, as opposed to pregnancy of which half are not planned and often go unnoticed for a period.

Studies about calls from pregnant women to medicines information or teratology centres, has shown that most calls regarded exposures in the first trimester [24, 25, 28]. This is somewhat in contrast to our findings, where questions from pregnant women most frequently concerned exposures in the second trimester. On the other hand, women in the first and second trimesters of pregnancy are shown to perceive significantly higher risk of medication use [32]. Altogether, we found that questions to SafeMotherMedicine reflected that women in all stages of pregnancy and breastfeeding have an information need about use of medications, an important aspect to keep in mind when developing medicines information to this patient group.

### Questions about breastfeeding

The number of questions concerning use of medications during breastfeeding (42%) was somewhat higher in our material compared to what is reported from other TIS (34–39%) [24, 27, 28]. Norway has a well-established and strong culture for breastfeeding and are among the countries with both the highest rate and duration of breastfeeding [33–35]. Additionally, a long paid maternity leave supports the possibility of breastfeeding throughout the first year [33]. The duration of breastfeeding in Norway, may be reflected in the finding that among the questions where the age of the breastfed child was known, we found more questions concerning breastfeeding of children above 12 months of age, than for newborns aged 0–2 weeks. The possibility of breastfeeding may not be addressed when medications are prescribed to a woman who gave birth more than one year ago, but our results illustrate an information need also among these women.

### Safety advice provided

Information about safety of medications in pregnancy and breastfeeding is available from multiple sources, and particularly online information has been shown to be frequently used by pregnant women [12, 36], despite being conflicting and inaccurate in many cases [11, 13, 14]. Thus, it can be challenging for pregnant and breastfeeding women to decide which information to trust. In addition, presence of conflicting information has been associated with sub-optimal use of medications and increased anxiety [14, 15]. The importance of having a medicines information service available to pregnant and breastfeeding women is underscored by the finding that SafeMotherMedicine provided reassuring advice with regard to all questions where either use of medications was discouraged by other sources, or that the information in other sources was conflicting. Moreover, avoidance of medications was recommended in only a few cases, in line with a study showing that advice from medicines information centres (RELIS) commonly were less restrictive than product information [26]. We found no pattern with regard to which medicines were associated with conflicting information on safety, which suggests that the challenge with inconsistent information about safety of use of medications during pregnancy and breastfeeding is a general issue, which does not concern particular medications or group of medications.

Pregnant and breastfeeding women are among patient groups where there has been a focus on increasing the digital health literacy, which is the ability to find, understand, and appraise health information from electronic sources [37]. By offering pregnant and breastfeeding women a web-based medicines information service, they are met in one of their preferred arenas for information seeking with high quality information, which the women further can discuss with their healthcare professionals.

### Strengths and limitations

Few studies on web-based medicines information services to pregnant and breastfeeding women have been conducted, and this study is the first to systemize spontaneous questions about use of medications from pregnant and breastfeeding women in Norway, to identify the information need in this patient group.

A limitation of the study is the lack of demographic data of the users of the service, which complicates the evaluation of the generalisability of the results. Another limitation is that information on stage of pregnancy and age of breastfed infant at time of use, and reason for raising a question to SafeMotherMedicine was not mandatory in the question form, and thus is missing in some of the questions. Lastly, questions to the service are individual and spontaneous, and does not necessary reflect questions from the general population of pregnant and breastfeeding women. However, the number of unique users on the SafeMotherMedicine webpage (approximately 45 000 users/year) compared to the number of births (approximately 55 000 births/year [38], suggests that a large proportion of the target population is familiar with the service. SafeMotherMedicine is the only available medicines information service directed at pregnant and breastfeeding women in Norway, which implies that our findings reflect the information need on the Norwegian population level.

### Future perspectives

The identified target areas for medicines information efforts will be used for developing general information texts such as FAQ, available at the SafeMotherMedicine webpage. Furthermore, it would be of interest to study how web-based information and advice on benefit and risks of medications is perceived among pregnant and breastfeeding women to identify areas of quality improvement of such written information, and the impact of the advice given with respect to appropriate medication use. Furthermore, preferences with regard to alternative platforms for risk communication about medications (e.g. telephone, web-service, chat, or app) needs to be studied among both users and non-users of the service. Sociodemographic information about users of SafeMotherMedicine has not been registered as the service is confidential, and it would be of interest to conduct a survey to compare the users of the service with the non-users.

## Conclusions

The identified target areas for medicines information directed at pregnant and breastfeeding women included both symptomatic relief of common complaints, such as pain, nausea, and rhinitis, as well as treatment of chronic conditions such as allergy, psychiatric disorders, and asthma. Our findings imply that developing medicines information directed at the identified target areas, tailored to apply to all stages of pregnancy or breastfeeding, will meet the information need of a large proportion of this patient group. The findings of this study could guide future development of medicines information to pregnant and breastfeeding women.

## Supplementary Information

**Additional file** 1. 

**Additional file** 2.

**Additional file** 3. 

**Additional file** 4.

## Data Availability

The data that support the findings of this study are available from the SafeMotherMedicine database, owned by Regional Medicines Information and Pharmacovigilance Centres, Norway. Restrictions apply to the availability of these data, which were used under license for the current study, and they are not publicly available. Data are however available from the authors upon reasonable request.

## Abbreviations

RELIS: Regional Medicines Information and Pharmacovigilance Centres, Norway
ATC: Anatomical therapeutic chemical classification system
SQL: Structured Query Language
ICC: Intraclass Correlation Coefficient
OTC: Over-the-counter
TIS: Teratology information services
