# Supplementary material for: Identifying target areas of medicines information efforts to pregnant and breastfeeding women by reviewing questions to SafeMotherMedicine: A Norwegian web-based public medicines information service
Source: BMC Pregnancy Childbirth. 2022 Dec 2;22:893. doi: 10.1186/s12884-022-05252-3 (PMC9717428; doi:10.1186/s12884-022-05252-3)
Supplement: Supplementary file 1 — Additional file 1. [file 12884_2022_5252_MOESM1_ESM.pdf]

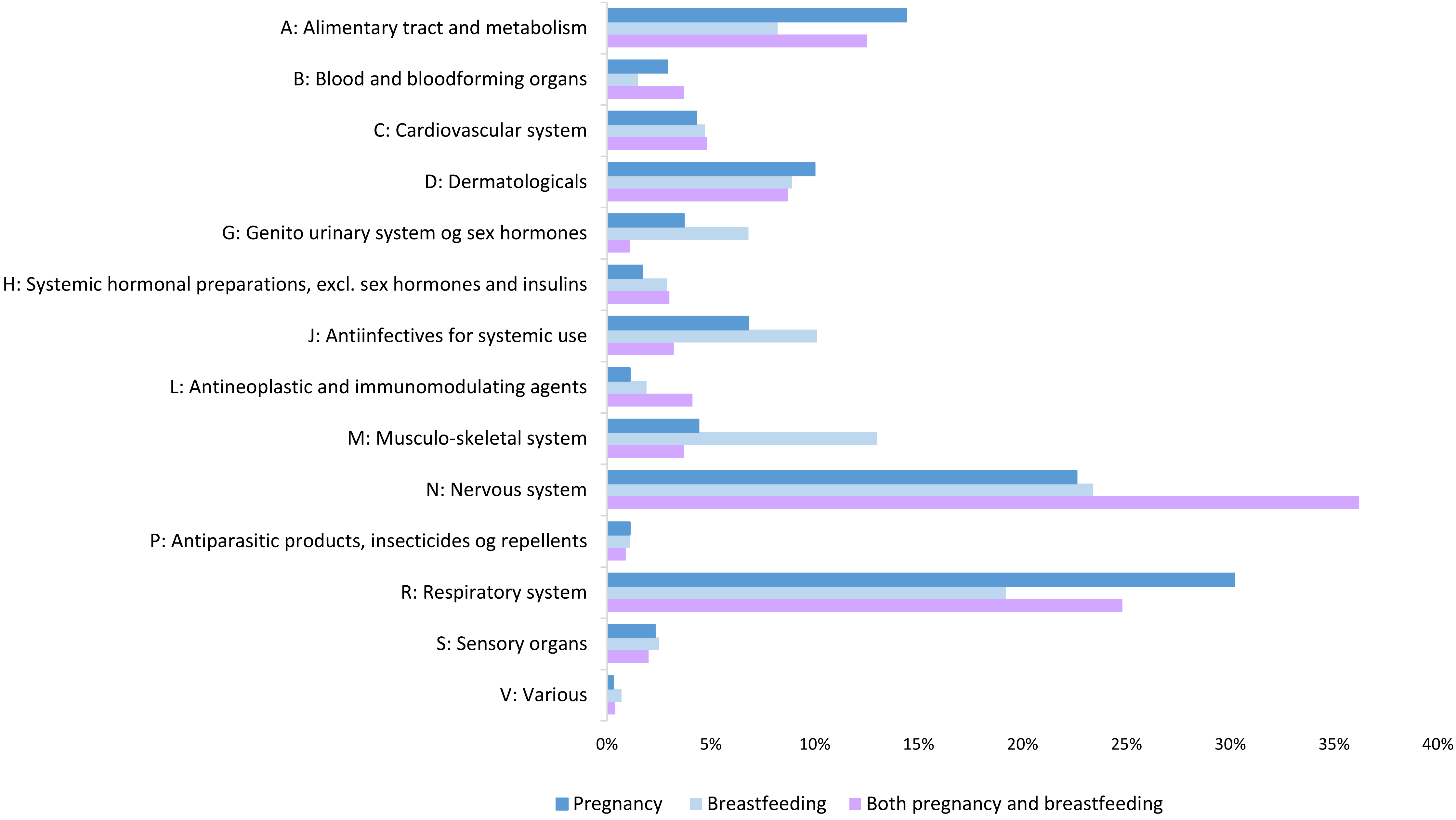

Supplementary Figure 1. Distribution of medications according to main ATC-groups

Distribution of main ATC-groups (ATC 1st level) among questions concerning medications in either pregnancy (n=5 985), breastfeeding (n=4 878) or both pregnancy and breastfeeding (n=755) to SafeMotherMedicine from January 2016 to September 2018. The ATC 1st level has 14 main anatomical or pharmacological groups. Notice that each question can include several medications and ATC-codes, and the sum exceeds 100%.
