# Supplementary material for: Identifying target areas of medicines information efforts to pregnant and breastfeeding women by reviewing questions to SafeMotherMedicine: A Norwegian web-based public medicines information service
Source: BMC Pregnancy Childbirth. 2022 Dec 2;22:893. doi: 10.1186/s12884-022-05252-3 (PMC9717428; doi:10.1186/s12884-022-05252-3)
Supplement: Supplementary file 2 — Additional file 2. [file 12884_2022_5252_MOESM2_ESM.pdf]

**Supplementary Table 1. Top 20 medications for use during pregnancy**

Top 20 medications most frequently asked about concerning use during pregnancy (n=5 985). Data based on questions to SafeMotherMedicine from January 2016 to September 2018.

| ATC-code | Substance | Therapeutic field | Number of questions n (%) |
|---|---|---|---|
| N02BE01 | Paracetamol | Pain | 441 (7.4) |
| R06AE05 | Meclizine | Nausea | 240 (4.0) |
| R06AE07 | Cetirizine | Allergy | 237 (4.0) |
| R01AA07 | Xylometazoline | Rhinitis | 218 (3.6) |
| R06AX27 | Desloratadine | Allergy | 199 (3.3) |
| G01AF02 | Clotrimazole (vaginal use) | Vaginal yeast infection | 149 (2.5) |
| C05AA04 | Prednisolone and dibucaine | Haemorrhoids | 140 (2.3) |
| N02CC01 | Sumatriptan | Migraine | 123 (2.1) |
| M01AE01 | Ibuprofen | Pain | 114 (1.9) |
| A03FA01 | Metoclopramide | Nausea | 112 (1.9) |
| D01AC01 | Clotrimazole (topical use) | Vaginal yeast infection | 110 (1.8) |
| N02AJ06 | Codeine and paracetamol | Pain | 110 (1.8) |
| R06AD02 | Promethazine | Nausea | 110 (1.8) |
| R03AC02 | Salbutamol | Asthma | 108 (1.8) |
| R01AD09 | Mometasone (nasal bruk) | Allergy | 104 (1.7) |
| R05CB02 | Bromhexine | Cough | 99 (1.7) |
| N05BA04 | Oksazepam | Anxiety/depression | 96 (1.6) |
| N06AB10 | Escitalopram | Anxiety/depression | 96 (1.6) |
| R01AA05 | Oksymetazoline | Rhinitis | 89 (1.5) |
| A04AA01 | Ondansetron | Nausea | 84 (1.4) |
