# Supplementary material for: Identifying target areas of medicines information efforts to pregnant and breastfeeding women by reviewing questions to SafeMotherMedicine: A Norwegian web-based public medicines information service
Source: BMC Pregnancy Childbirth. 2022 Dec 2;22:893. doi: 10.1186/s12884-022-05252-3 (PMC9717428; doi:10.1186/s12884-022-05252-3)
Supplement: Supplementary file 3 — Additional file 3. [file 12884_2022_5252_MOESM3_ESM.pdf]

Supplementary Table 2. Top 20 medications for use during breastfeeding

Top 20 medications most frequently asked about concerning use during breastfeeding (n=4 878). Data based on questions to SafeMotherMedicine from January 2016 to September 2018.

ATC-code	Substance	Therapeutic field	Number of questions n (%)
M01AE01	Ibuprofen	Pain	356 (7.3)
N02BE01	Paracetamol	Pain	351 (7.2)
M01AB05	Diclofenac	Pain	168 (3.4)
N02AJ06	Codeine and paracetamol	Pain	140 (2.9)
R06AX27	Desloratadine	Allergy	131 (2.7)
R06AE07	Cetirizine	Allergy	110 (2.3)
J01CF01	Dicloxacillin	Mastitis	107 (2.2)
N02CC01	Sumatriptan	Migraine	107 (2.2)
D01AC01	Clotrimazole (topical use)	Vaginal yeast infection	97 (2.0)
N06AB10	Escitalopram	Anxiety/depression	83 (1.7)
C05AA04	Prednisolone and dibucaine	Haemorrhoids	81 (1.7)
R01AA07	Xylometazoline	Rhinitis	81 (1.7)
R05DA07	Noscapine	Cough	74 (1.5)
M02AA15	Diclofenac (topical use)	Pain	67 (1.4)
R05DA01	Ethylmorphine	Cough	64 (1.3)
R06AE05	Meclizine	Nausea	62 (1.3)
N02AX02	Tramadol	Pain	61 (1.3)
M02AA13	Ibuprofen (topical use)	Pain	59 (1.2)
R05CB02	Bromhexine	Cough	59 (1.2)
R06AX13	Loratadine	Allergy	58 (1.2)
