# Supplementary material for: Identifying target areas of medicines information efforts to pregnant and breastfeeding women by reviewing questions to SafeMotherMedicine: A Norwegian web-based public medicines information service
Source: BMC Pregnancy Childbirth. 2022 Dec 2;22:893. doi: 10.1186/s12884-022-05252-3 (PMC9717428; doi:10.1186/s12884-022-05252-3)
Supplement: Supplementary file 4 — Additional file 4. [file 12884_2022_5252_MOESM4_ESM.pdf]

Supplementary Table 3. Top 20 medications for use during both pregnancy and breastfeeding

Top 20 medications most frequently asked about concerning use during both pregnancy and breastfeeding (n=755). Data based on questions to SafeMotherMedicine from January 2016 to September 2018.

ATC-code	Substance	Therapeutic field	Number of questions n (%)
N06AB10	Escitalopram	Anxiety/depression and other psychiatric disorders and symptoms	50 (6.6)
R06AX27	Desloratadine	Allergy	42 (5.6)
N05AH04	Quetiapine	Anxiety/depression and other psychiatric disorders and symptoms	35 (4.6)
R06AE07	Cetirizine	Allergy	34 (4.5)
R06AE05	Meclizine	Nausea	23 (3.0)
N06AB06	Sertraline	Anxiety/depression and other psychiatric disorders and symptoms	21 (2.8)
N02BE01	Paracetamol	Pain	20 (2.6)
N03AX09	Lamotrigine	Bipolar disorder/epilepsy	19 (2.5)
N06AX16	Venlafaxine	Anxiety/depression and other psychiatric disorders and symptoms	18 (2.4)
N06BA04	Methylphenidate	ADHD	17 (2.3)
R03AC02	Salbutamol	Asthma	17 (2.3)
A07EC02	Mesalamine	Inflammatory bowel disease	16 (2.1)
A03FA01	Metoclopramide	Nausea	14 (1.9)
N02CC01	Sumatriptan	Migraine	14 (1.9)
R01AD09	Mometasone (nasal use)	Allergy	14 (1.9)
R01AA07	Xylometazoline	Rhinitis	13 (1.7)
N05CH01	Melatonine	Insomnia	12 (1.6)
R06AX13	Loratadine	Allergy	12 (1.6)
N06AB03	Fluoxetine	Anxiety/depression and other psychiatric disorders and symptoms	11 (1.5)
A02BC05	Esomeprazole	Gastrointestinal reflux	10 (1.3)
L04AX01	Azathioprine	Inflammatory bowel disease	10 (1.3)
N02CC04	Rizatriptan	Migraine	10 (1.3)
R03AK07	Formoterol and budesonide	Asthma	10 (1.3)
